# PPARα: A potential therapeutic target of cholestasis

**DOI:** 10.3389/fphar.2022.916866

**Published:** 2022-07-18

**Authors:** Xiaoyin Ye, Tong Zhang, Han Han

**Affiliations:** ^1^ School of Traditional Chinese Medicine, Shanghai University of Traditional Chinese Medicine, Shanghai, China; ^2^ Experiment Center for Teaching and Learning, Shanghai University of Traditional Chinese Medicine, Shanghai, China

**Keywords:** cholestasis, inflammation, liver injury, peroxisome proliferator-activated receptor alpha, therapeutic target

## Abstract

The accumulation of bile acids in the liver leads to the development of cholestasis and hepatocyte injury. Nuclear receptors control the synthesis and transport of bile acids in the liver. Among them, the farnesoid X receptor (FXR) is the most common receptor studied in treating cholestasis. The activation of this receptor can reduce the amount of bile acid synthesis and decrease the bile acid content in the liver, alleviating cholestasis. Ursodeoxycholic acid (UDCA) and obeticholic acid (OCA) have a FXR excitatory effect, but the unresponsiveness of some patients and the side effect of pruritus seriously affect the results of UDCA or OCA treatment. The activator of peroxisome proliferator-activated receptor alpha (PPARα) has emerged as a new target for controlling the synthesis and transport of bile acids during cholestasis. Moreover, the anti-inflammatory effect of PPARα can effectively reduce cholestatic liver injury, thereby improving patients’ physiological status. Here, we will focus on the function of PPARα and its involvement in the regulation of bile acid transport and metabolism. In addition, the anti-inflammatory effects of PPARα will be discussed in some detail. Finally, we will discuss the application of PPARα agonists for cholestatic liver disorders.

## Introduction

Bile acids are synthesized from cholesterol in the liver, secreted in the bile, and carried to the intestines (Di Ciaula et al., 2017). In other words, bile acids are the products of cholesterol. Hepatocyte dysfunction results in bile acid accumulation in the liver and occurrence of cholestasis. Cholestasis can be caused by the mechanical blockage of the bile duct (Chai et al., 2015), gene defect (Bull and Thompson, 2018), hormonal disorders (Piechota and Jelski, 2020), and drug administration (Gijbels et al., 2019). Generally speaking, cholestasis can be categorized into two types according to the location of the bile flow disturbance: intrahepatic and extrahepatic cholestasis. Alkaline phosphatase (ALP), γ-glutamyl transpeptidase (GGT), 5′-nucleotidase, aspartate aminotransferase, and alanine aminotransferase (ALT) are used as indicators for diagnosing cholestasis (Padda et al., 2011).

Although the clinical manifestations of cholestasis are diverse, all of them have a common feature: excess bile acids accumulate in the liver and cause hepatocyte damage (Halilbasic et al., 2015; Patel and Seetharam, 2016). Meanwhile, nuclear receptors are critical components involved in the regulation of bile acid transporters. They have been used as therapeutic targets for cholestatic liver diseases. The farnesoid X receptor (FXR) is a bile acid-activated transcription factor and is essential for bile acid homeostasis (Keitel et al., 2019). FXR is mainly expressed in the liver and intestine and controls cholestasis by sensing bile acids and regulating them through negative feedback (Forman et al., 1995; Cariello et al., 2018; Ticho et al., 2019). Current treatments are all associated with FXR, including ursodeoxycholic acid (UDCA), which partly affects FXR (Li et al., 2016), and obeticholic acid (OCA), which is a direct FXR agonist. For example, FXR agonists and OCA are recommended to use in patients with primary biliary cholangitis (PBC) (Lindor et al., 2009; Gulamhusein and Hirschfield, 2020). Despite advances in cholestasis treatment, it has unwanted side effects, including pruritus, worsening liver function, headache, and anemia (D'Amato et al., 2021). In the clinic, UDCA is ineffective for most patients, and its narrow application range is only useful to patients with PBC (Leuschner et al., 2000). Meanwhile, OCA may cause pruritus in some patients, preventing further treatment (Younossi et al., 2019). Even worse, some studies also proved that FXR activation may aggravate obstructive cholestasis (Stedman et al., 2006). Therefore, expanding our knowledge of cholestatic liver injury and finding novel nuclear receptors and medical treatments to treat cholestasis without side effects are necessary. In this review, we provide an overview of the function of peroxisome proliferator-activated receptor alpha (PPARα) and its adaptive response to cholestasis. PPARα may serve as a therapeutic target for the treatment of cholestatic disorders.

## Burden of cholestasis-exceed bile acid and inflammation

In the pathological process of cholestasis, bile acids accumulate in the liver due to transporter disorders. The symptom may develop into cirrhosis or liver failure and subsequently result in liver transplantation. Bile acids are considered the direct reasons for hepatocyte damage. Chenodeoxycholate is highly toxic and causes hepatocyte injury (Spivey et al., 1993). However, this burden of cholestasis is complex. We have summarized it in Table 1.

**TABLE 1:** The potential mechanisms of hepatocytes injury during cholestasis.

The cause of hepatocytes injury	Outcomes	Mechanisms
Bile acid cytotoxicity	Hepatocytes apoptosis, Inflammation	Chenodeoxycholate depletes ATP and lead to the lethal cell injury of anoxia. The secondary bile acid, lithocholic acid, can also cause damage to liver cells
Inflammation	Hepatocytes necrosis, Fibrosis	a.Bile acids active Egr-1 resulting in neutrophil accumulation
		b.Bile acid induces ATP releasing K^+^ activing inflammasome
		c.Injured hepatocytes release mtDNA and detected by toll-like receptor 9 which can attract chemokines
Fibrosis	Cirrhosis, Liver failure	Neutrophils induce oxidative stress to injure hepatocytes lead to fibrosis

Intrahepatic cholestasis is a disease that often occurs during pregnancy and is accompanied by pruritus or elevated serum transaminases. At the same time, the bile acid content in the blood also increases. This symptom not only affects pregnant women but also causes complications for newborns, which may lead to their death (Floreani and Gervasi, 2016). Progressive familial intrahepatic cholestasis (PFIC) is a genetic disease caused by a genetic defect. Three main subtypes of PFIC have been identified. Patients show jaundice and pruritus in infancy or early childhood. This will induce a series of poor outcomes including cirrhosis and liver failure (Baker et al., 2019). Newborns usually have neonatal jaundice and even neonatal cholestasis. This may lead to abnormal liver function, liver failure, and even death (Satrom and Gourley, 2016). In conclusion, cholestasis can cause damage to the liver, leading to liver failure and cirrhosis. Clinically, it will be characterized by an increase in the total bile acid content in the blood. If patients cannot get effective treatment, then liver transplant will be the only choice for them.

Meanwhile, it should be noted that inflammation is an important reason for liver injury. Necrosis is more common than apoptosis in the area of cholestatic liver injury, which is a standard inflammatory feature (Woolbright and Jaeschke, 2012). After 6 h of bile duct ligation (BDL), neutrophils will accumulate in the area of necrosis and liver injury (Woolbright et al., 2013). Bile acids kill hepatocytes by activating neutrophils to produce reactive oxygen species. Inhibiting neutrophil function in hepatocytes can reduce oxidative stress and liver injury (Copple et al., 2010). The expression levels of serum inflammatory cytokines, such as tumor necrosis factor-α (TNF-α), interleukin (IL)-1β, and IL-6, are increased in patients with cholestasis, demonstrating that inflammation plays a role in cholestasis (Barak et al., 2009). Corilagin reduces cholestatic liver injury induced by alpha-naphthylisothiocyanate by exerting anti-inflammation effects and decreasing nuclear factor kappa-B (NF-κB) levels (Jin et al., 2013). Stearic acid, a drug with anti-inflammatory potential, also attenuates the pathophysiological changes in cholestasis induced by BDL (Pan et al., 2010). The hepatoprotective effect of stearic acid is associated with anti-inflammatory effects (Pan et al., 2010).

**FIGURE 1:**
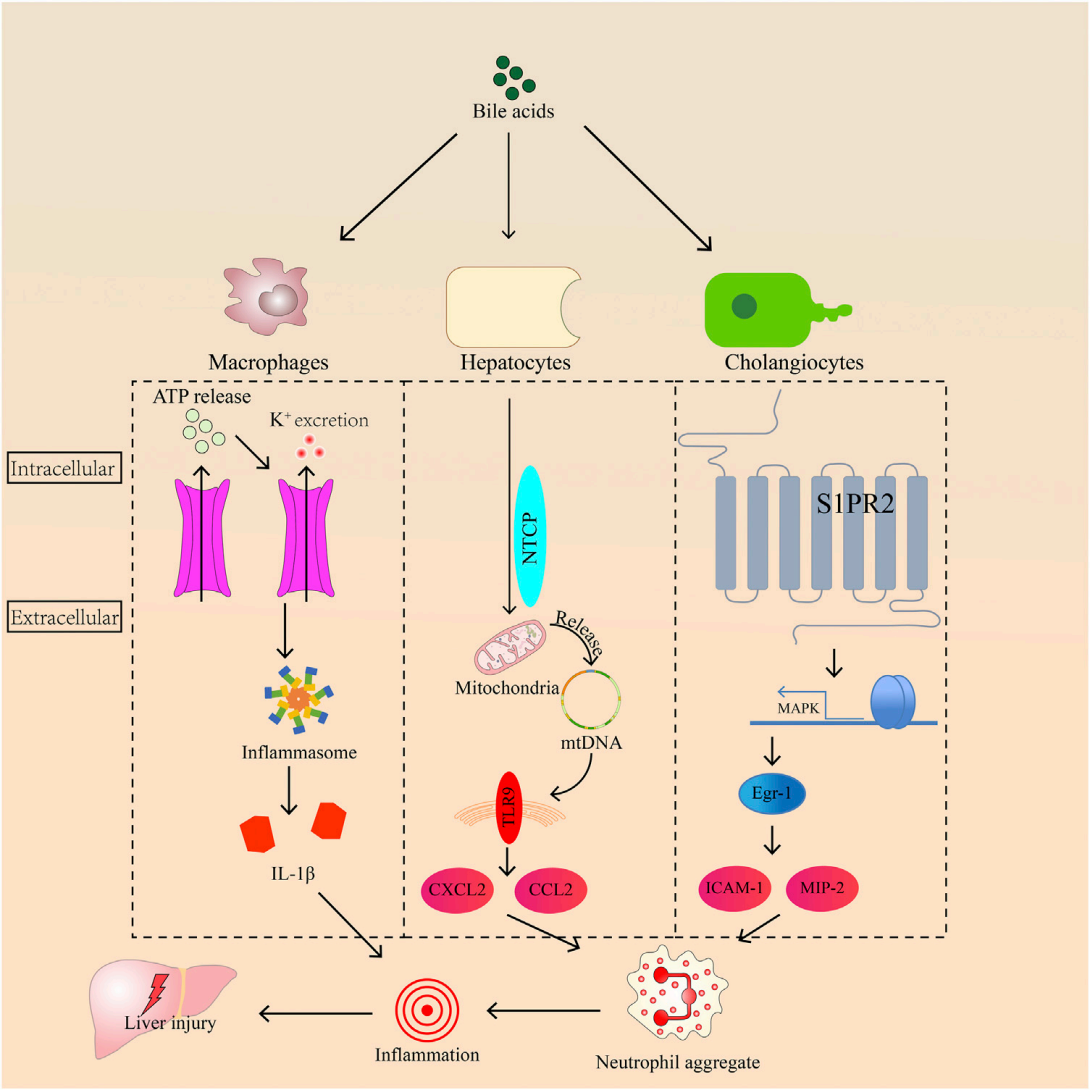
The roles of Egr-1, toll-like receptor-9, and inflammasome in regulating inflammation induced by bile acids in hepatocyte.

Therefore, we consider that inflammation is an inevitable burden of cholestasis. The mechanism of bile acids in triggering inflammation remains controversial. Early growth response factor-1 (Egr-1), inflammasome, and Toll-like receptors are all related to inflammation caused by bile acid (Figure 1). Three pathways will be described in detail as follows.

Egr-1 plays an important role in the development of inflammation during cholestasis. It is activated by bile acids in hepatocytes, and its activation requires mitogen-activated protein kinase (MAPK) signaling. Then, Egr-1 can activate intercellular adhesion molecule 1 (ICAM-1) production, and macrophage inflammatory protein 2 induces neutrophil aggregation, leading to inflammation (Kim et al., 2006; Allen et al., 2010). Sphingosine-1-phosphate receptor 2 (S1PR2), a bile acid sensor, transmits signals to bile acids and Egr-1 through MAPK, resulting in neutrophil accumulation in the liver (Zhang et al., 2019). Inflammasomes consist of an upstream sensor protein of the NOD-like receptor family, an adaptor protein, and the downstream effector protease caspase-1 (de Vasconcelos et al., 2016). The inflammasome is activated by K^+^ outflow caused by bile acids in macrophages, inducing ATP release (Gong et al., 2016). The autocatalytic cleavage of caspase-1 promotes proinflammatory factor pro-IL-1β and pro-IL-18 maturity when sensor receptors are stimulated by microbial or sterile stressors (Elliott and Sutterwala, 2015). Meanwhile, bile acids activate inflammasomes in inflammatory macrophages (Hao et al., 2017). For example, chenodeoxycholic acid increases the concentrations of IL-1β through the activation of the NLRP3 inflammasome in macrophages (Gong et al., 2016). Toll-like receptors, protective immune sentries, sense pathogen- or damage-associated molecular patterns and trigger gene expression changes that ultimately eradicate invading microbes (Lim and Staudt, 2013). *In vitro*, high levels of bile acids injure the mitochondria by changing their membrane potential and releasing cytochrome c (Botla et al., 1995). Patients with cholestasis have high bile acid levels in liver cells. This symptom may also cause mitochondrial damage in the hepatocytes, damaging or releasing the mitochondrial genome (mtDNA) from mitochondria. Some studies also proved that cholestasis is often accompanied by mtDNA damage (Tiao et al., 2007; Xu et al., 2012). In hepatocytes, the mitochondria release mtDNA, which can be detected by Toll-like receptor 9. Ultimately, bile acids induce cell necrosis and release C-X-C motif chemokine ligand 2 and chemokine ligand 2 to recruit neutrophils that cause inflammation through damaged mtDNA (Cai et al., 2017).

In addition to causing liver injury, inflammation can aggravate cholestasis by affecting transporters of bile acids in hepatocytes. TNF-α and IL-1 can reduce the mRNA expression levels of bile salt export pump (BSEP) and multidrug resistance-associated protein 2 (MRP2), which control bile acid discharge from the liver (Azeltine et al., 2020). Meanwhile, oxidative stress caused by neutrophils is a mechanism of cell death, and subsequent fibrosis results in cirrhosis and liver failure. Thus, controlling inflammation is as important as regulating the size of the bile acid pool in cholestasis treatment.

## Peroxisome proliferator-activated receptor alpha

Peroxisome proliferator-activated receptors (PPARs) belong to the subfamily 1 of the nuclear hormone receptor superfamily of transcription factors (Nuclear Receptors Nomenclature Committee, 1999) and regulate genes important for cell differentiation and various metabolic processes, especially lipid and glucose homeostasis (Grygiel-Gorniak, 2014). PPARs comprise the following three subtypes: PPARα, PPARγ, and PPARδ (also designated as PPARβ). PPARs have the basic structural properties of most nuclear receptors; that is, PPARs consist of four functional domains, namely, A/B, C, D, and E/F. The N-terminal A/B domain contains the ligand-independent activation function 1, which is responsible for PPAR phosphorylation. The conserved central DNA binding domain, also known as the C domain, is composed of two zinc fingers and is responsible for the binding of PPAR to the peroxisome proliferator response element (PPRE) in the promoter of the PPAR target genes. The D domain is a docking site for various cofactors. The E domain is also named the ligand-binding domain (Christofide s et al., 2021). The full transcriptional activity of PPARs requires the binding of cognate lipid ligands and heterodimerization with another nuclear receptor, retinoid-X receptor (RXR) (Gyamfi and Wan, 2009). PPARs stimulate the expression of several genes by binding to specific PPREs through cooperation with retinoid X receptors.

PPARα is expressed in the skeletal muscles, heart, liver, kidney, and brown adipose tissues and is associated with fatty acid catabolism (Han et al., 2020). PPARα agonists can control bile acid homeostasis by inducing metabolic enzymes and inhibiting bile acid synthesis (Ghonem et al., 2015). The expression of PPARα is associated with fatty acid catabolism, and PPARα functions as a lipid sensor and controls energy combustion (Ip et al., 2003; Han et al., 2020). Meanwhile, PPARα plays a vital role in glucose homeostasis and insulin resistance development (Fruchart et al., 2001). Synthetic PPARα agonists have been identified, including fenofibrate, which is one of the most commonly used fibrates in cholestasis treatment trials (Dai et al., 2017); WY-14643; and bezafibrate, which is a pan-PPAR agonist. The natural ligands of PPARα mainly include unsaturated fatty acids, leukotriene B4, and 8-hydroxyeicosatetraenoic acid (Grygiel-Gorniak, 2014). Normally, PPARα agonists are used to treat patients with fatty liver, diabetes, and dyslipidemia (Oscarsson et al., 2018; Zhu et al., 2020). Recently, researchers found that PPARα agonists can improve the condition of patients with primary sclerosing cholangitis (PSC) and PBC (de Vries et al., 2021). Meanwhile, another study had shown that they can be used as a treatment for cholestasis (Honda et al., 2013).

## Regulation of bile acid transport and metabolism by peroxisome proliferator-activated receptor alpha

**FIGURE 2:**
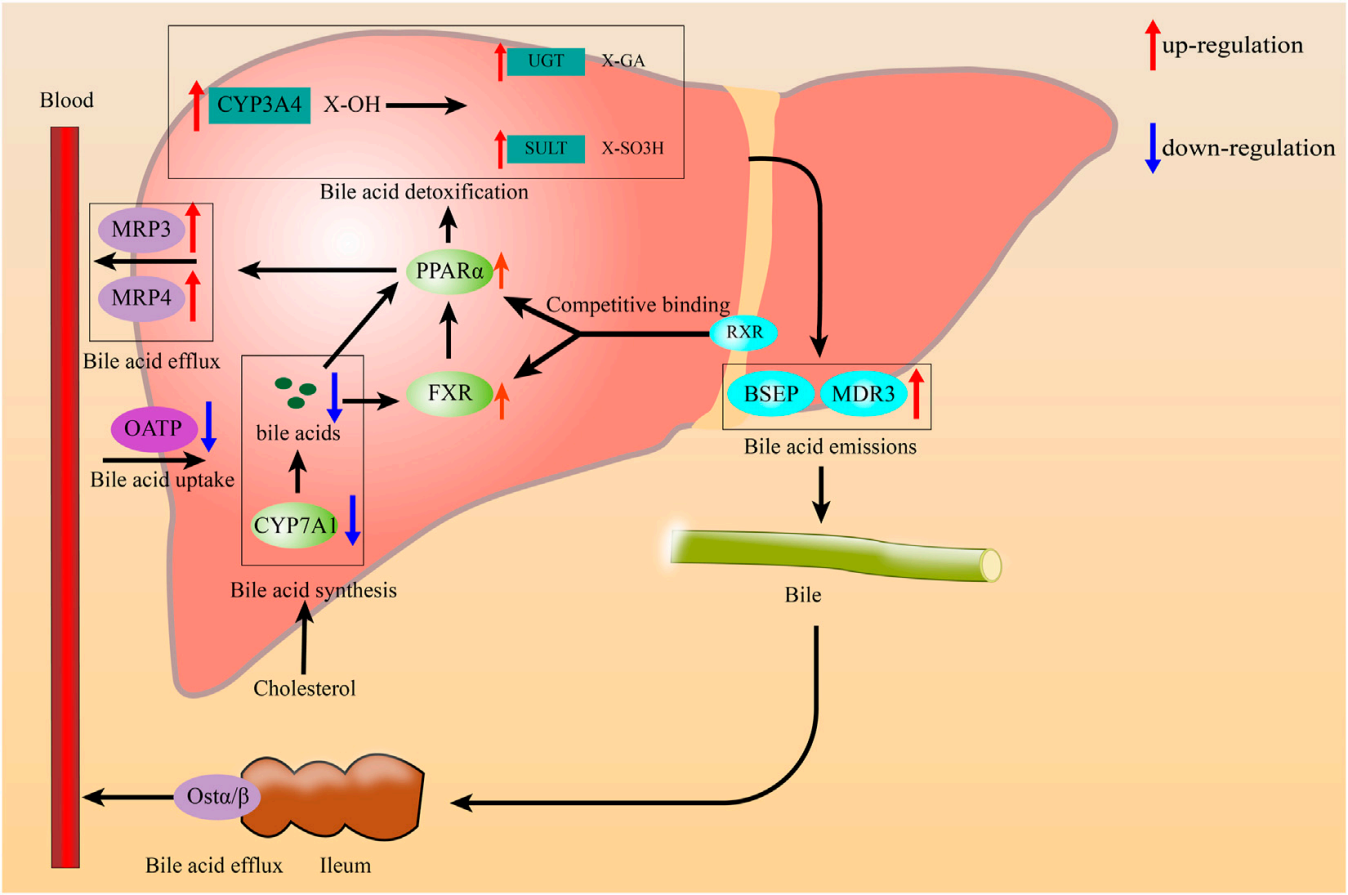
The role of PPARα in reguleation bile acids transport and metabolism.

*In vivo*, the activation of PPARα is associated with the increased hepatobiliary circulation of bile acids, inhibition of hepatic bile acid biosynthesis, and reduction in plasma triglycerides (Zollner et al., 2010). We will discuss the roles of PPARα in bile acid transport and metabolism and its implications for cholestatic disorders (Figure 2).

### Activation of peroxisome proliferator-activated receptor alpha by bile acids

As a nuclear receptor that controls lipid and glucose metabolism, PPARα can be influenced by bile acid. In addition to activated bile acids, PPARα is a transcriptional target of bile acid-activated FXR, indicating that it can be indirectly activated by bile acids through FXR (Pineda et al., 2003; Dai et al., 2018; Hua et al., 2019). However, when mice were fed with a bile acid-enriched diet, PPARα was inhibited by bile acids (Sinal et al., 2001). The mRNA levels of targets for PPARα, including CYP4A1, CYP4A3, ACOX, BE, and thiolase, were reduced substantially when using combined cholic acid/WY-14643 diet compared with WY-14643 alone. Moreover, bile acids control intestinal peptide absorption transporter in the gut by inhibiting PPARα (Okamura et al., 2014). Although bile acids can indirectly activate PPARα through FXR, the real relationship between bile acids and PPARα is inhibition.

### Regulation of bile acid metabolism

The activation of PPARα by ligands, including WY-14643 and fatty acids, or during fasting induces a range of actions involved in bile acid homeostasis. PPARα detoxification pathways that process harmful bile acids can be mainly divided into two types, namely, hydroxylation by members of the cytochrome P450 subfamily and conjugation by UDP glucuronosyltransferases (UGTs) and sulfotransferases (SULTs). These processes reduce the number of harmful bile acids in the body by inhibiting the generation of bile acids, increasing the polarity of bile acids, and facilitating excretion through bile or urine. Cholesterol 7 alpha-hydroxylase (CYP7A1), a rate-limiting enzyme of bile acid production, is downregulated by PPARα. Treatment with 0.05% ciprofibrate decreased CYP7A1 enzyme activity and mRNA, but these effects were completely abolished in Pparα^−/−^ mice (Patel et al., 2000; Post et al., 2001). Meanwhile, sterol 26-hydroxylase (CYP27A1), the key enzyme of alternate pathways of bile acid synthesis, is also reduced in this case (Post et al., 2001). The inhibition of murine Cyp7a1 gene caused by PPARα activation is sensitive to the human CYP7A1 gene. The differential response of the murine Cyp7a1 and human CYP7A1 gene promoters to PPARα activators is attributable to the additional PPARα/RXRα-binding site in the murine CYP7A1 gene promoter (Cheema and Agellon, 2000). After HepG2 cells were cultured with WY-14643, the level of CYP7A1 transactivation decreased with HNF-4 alpha level. This result suggested that PPARα and agonist repress CYP7A1 by reducing the availability of HNF-4 that would bind the DR-1 sequence, thereby attenuating CYP7A1 transactivation by HNF-4 (Marrapodi and Chiang, 2000). In addition to CYP7A1, PPARα can activate other cytochromes to promote bile acid excretion out of the body. Perfluorodecanoic acid is a persistent organic pollutant with the ability to activate PPARα. Studies found that perfluorodecanoic acid reduced the mRNA level of Cyp7a1, Cyp8b1, and sodium taurocholate cotransporting polypeptide (NTCP) in mice administered with perfluorodecanoic acid. This phenomenon was not found in Pparα-null mice (Luo et al., 2017). The reduction of the above three proteins can reduce the production of bile acid in the liver and reduce the size of the bile acid pool of hepatocytes.

CYP3A4 is the major enzyme expressed in the liver and gut. It is involved in the detoxification, hydrolyzation, and subsequent glucuronidation of bile acids by UGTs (Chen et al., 2014). *In vivo*, WY-14643 is involved in the induction of CYP3A4 mRNA in the liver but not in the intestine; this finding establishes PPARα as a direct transcriptional regulator of hepatic CYP3A4 (Thomas et al., 2013). Sterol 12α-hydroxylase (CYP8B1) increases the level of cholic acid/chenodeoxycholic acid. Chenodeoxycholic acid is converted into lithocholic acid after 7α-dehydroxylation by coliform flora. Therefore, the activation of CYP8B1 can decrease the production of lithocholic acid to reduce the hepatotoxicity of total bile acids in hepatocytes. The expression of Cyp8b1 decreased in stard10^−/−^ mice after PPARα activity was impaired (Ito et al., 2013). PPARα activation can induce an increase in taurocholic acid level and is related to an increase in CYP8B1 level (Xie et al., 2019). *In vitro*, WY-14643 treatment increased the relative amount of cholic acid in HepG2 cells by activating CYP8B1 (Shi et al., 2005). PPRE identified in the rat sterol 12 alpha-hydroxylase promoter region in HepG2 cells was activated after WY-14643 treatment (Hunt et al., 2000). Therefore, PPARα can decrease the toxicity of total bile acids by decreasing the level of chenodeoxycholic acid. Other cytochromes, such as cytochrome P450 3a, cytochrome P450 2b, and cytochrome P450 2c, were induced in a dose-dependent manner by gemfibrozil (Shi et al., 2017).

Organic anion-transporting polypeptide (OATP) mediates the Na^+^-independent transport of organic anions such as sulfobromophthalein and conjugated and unconjugated bile acids to the liver. *In vivo*, the mRNA expression of Oatp1a1, 1b2, 2a1, and 2b1 in the liver is decreased by PPARα ligands (clofibrate, ciprofibrate, and diethylhexylphthalate) (Cheng et al., 2005). Through this process, the bile acid level in the liver was decreased.

The UGT family is responsible for the transfer of glucuronic acid to other molecules, such as bile acids, and acts as a catalyst. The induction of UGT2B4 by bile acids contributes to a feed-forward reduction of bile acid toxicity (Barbier et al., 2003a). The incubation of human hepatocytes with WY-14643 increases UGT2B4 mRNA levels (Barbier et al., 2003b). These results suggested that the activation of PPARα can reduce bile acid toxicity through UGT2B4. UGT1A1, UGT1A3, UGT1A4, and UGT1A6 are the targets of PPARα in human hepatocytes (Senekeo-Effenberger et al., 2007). SULT catalyzes the sulfation of bile acids, increases its water solubility, and promotes excretion. PPARα participates in the transcriptional regulation of SULT2A1 and SULT2A8 (Fang et al., 2005; Feng et al., 2017).

Organic solute transporter (OST) subunits OSTα and OSTβ facilitate bile acid efflux from the enterocyte into the portal circulation. OSTα/β knockout mice have longer and thicker small intestines and are largely protected against experimental cholestatic liver injury (van de Wiel et al., 2022). However, some researchers found that the level of OSTα/β expression was not changed in Oatp1a1-null BDL mice with increased Pparα expression (Zhang et al., 2012). We speculated that the ability of PPARα to regulate bile acids is not *via* OSTα/β.

### Regulation of bile acid elimination

Multidrug resistance 2 (MDR2, also known as ABCB4) is a multidrug resistance gene located in zone 1, region 2 of the long arm of chromosome 7. It mainly exists in the bile duct membranes of hepatocytes and is expressed in the normal human placenta. The MDR2 gene transfers phospholipids from hepatic lobules to the outer surface of the bile duct membrane (Elferink and Groen, 2002). Abcb4^−/−^ mice displayed progressive liver damage at an early age, and this effect was accompanied by hyperbilirubinemia and an increase in liver enzymes in the plasma (Elferink and Groen, 2002). Human MDR3 and mouse Mdr2 have a high degree of homology, and the p-gp amino acid sequences encoded by them have 90% similarity (Gros et al., 1988). Mdr2 plays an essential role in the secretion of phosphatidylcholine into bile and may be a phospholipid transport protein or phospholipid flippase (Smit et al., 1993). Phospholipids are essential components of the bile and reduce the detergent activity of bile acid micelles, thereby protecting the membranes of cells lining the biliary tree from damage. When Mdr2 is damaged, the amount of phospholipids in the bile ducts becomes insufficient, and liver damage subsequently occurs. PFIC3 is caused by the mutations in the ABCB4 gene (Davit-Spraul et al., 2010). In cholestasis, UDCA may contribute to therapeutic effects by inducing alternative excretory routes for bile acids and other cholephiles through activating ABCB4 (Zollner et al., 2003). Therefore, MDR3 can be a target for partial cholestasis treatment.

Fibrates, the agonists of PPARα, induce the hepatic expression of MDR2 and encode the canalicular phospholipid translocator (Kok et al., 2003). The secretion of phospholipids and cholesterol increased only during high-bile-salt infusions, and no fibrate effects were observed in PPARα^−/−^ mice. The exposure of cultured wild-type mouse hepatocytes to PPARα agonists specifically induced Mdr2 mRNA levels. Thus, PPARα increased the amounts of phospholipids in the canalicular network through Mdr2 in mice. However, given the species-specific nature of the gene, whether PPARα can activate ABCB4 in humans remains unclear. Thus, fenofibrate was used to stimulate human liver cells (Ghonem et al., 2014). Fenofibrate significantly upregulated MDR3 mRNA and protein expression in primary cultured human hepatocytes and stimulated MDR3 promoter activity in HepG2 cells. In silico analysis of the 5′-upstream region of the human MDR3 gene revealed a number of PPREs, showing that PPARα activates MDR3 gene transcription by directly binding to PPRE (Ghonem et al., 2014).

In addition to MDR3, BSEP and the multidrug resistance-associated protein family are associated with PPARα in bile acid homeostasis regulation. BSEP catalyzes the transport of major hydrophobic bile salts, such as taurine and glycine-conjugated cholic acid, across the canalicular membranes of hepatocytes in an ATP-dependent manner. Clofibrate, a PPARα agonist, reduced the total bile acids in mouse livers, but this effect was not observed in PPARα^−/−^ mice. An increase in the mRNA level of BSEP resulted in a reduction of total bile acids in livers (Zhang et al., 2017). Multidrug resistance-associated protein 3 (MRP3) and MRP4 are often adaptively upregulated in cholestasis and can partly alleviate bile acid accumulation in the liver. Thus, the upregulation of MRP3 and MRP4 may be an adjunct to the treatment of cholestasis. PPARα can regulate polysaccharide-resistant proteins (Moffit et al., 2006; Wang et al., 2018). For instance, the mRNA levels of MRP3 and MRP4 increased only in wild-type mice when wild-type and PPARα^−/−^ mice received clofibrate treatment (Moffit et al., 2006). All these proteins were found to be effective in inhibiting intrahepatic bile acid deposition.

### Crosstalk with nuclear farnesoid X receptor

FXR is the main regulator of bile acid homeostasis because it transcriptionally drives the modulation of bile acid synthesis, influx, efflux, and detoxification along the enterohepatic axis (Cariello et al., 2018). FXR belongs to the nuclear receptor family and is expressed in the liver; PPARα is activated in the liver by fatty acids during fasting (Pawlak et al., 2015), whereas FXR is activated by bile acid return in the liver during feeding (Preidis et al., 2017). The crosstalk between energy balance, including that between glucose and lipid, has been extensively explored (Preidis et al., 2017). We focus on the interaction between them in terms of bile acid homeostasis.

FXR decreases the rate of bile acid synthesis by activating the small heterodimer partner (SHP), thereby inhibiting CYP7A1, suppressing NTCP, and reducing the rate of bile acid absorption by hepatocytes while promoting the expression of BSEP and expelling bile acids from the liver (Ding et al., 2015). In addition, FXR counteracts liver X receptor in cholesterol and triglyceride metabolism (Kalaan y and Mangelsdorf, 2006). Thus, PPARα and FXR act on common metabolic pathways. The molecular crosstalk between these two nuclear receptors needs to be investigated.

The treatment of HepG2 cells with chenodeoxycholic acid led to a dose-dependent increase in hPPARα mRNA levels (Pineda et al., 2003). The induction of hPPARα expression by bile acids influenced the response of the PPARα target gene CPT-1 to PPARα ligands. This result suggested that an increase in PPARα expression occurs partly through the transcriptional mechanisms of FXR. The discovery of an FXR response element located in the human PPARα promoter further supports this standpoint. Therefore, activating FXR can upregulate the expression of PPARα. However, PPARα was found to have an inhibitory effect in rodents. 1-Naphthyl isocyanate (ANIT), a model drug for cholestasis, inhibits the expression of FXR (Zhang et al., 2020). When the control group and PPARα^−/−^ mice received 0.05% ANIT orally, the levels of Shp and Fxr mRNA doubled in the cholestatic PPARα^−/−^ mice compared with those in the control group (Hua et al., 2019). Similarly, the targets of PPARα were increased in Shp^−/−^ mice (Park et al., 2011). Meanwhile, a study found the relationship between PPARα and FGF15 (Zhou et al., 2014). FGF15/19 is also an important bile acid target gene regulated by FXR to control CYP7A1 upregulation. In the model of inflammatory bowel disease, the accumulation of bile acids in inflamed colon tissues can repress FXR-FGF15 signaling by activating the intestinal PPARα–UGT pathway to eliminate bile acids in the intestine. Treatment with PPARα agonist fenofibrate can decrease the level of serum concentrations of FGF-19 in obesity (Mraz et al., 2011). Thus, the crosstalk between basal PPARα and FXR occurred, and adaptation of bile acid metabolism was inhibited in chronic cholestasis (Hua et al., 2019). In addition to the indirect effects through regulation of bile acids, this crosstalk may be related to PPARα and FXR competing with RXRα (Xie et al., 2019). This research used chenodeoxycholic acid to activate FXR and WY-14643 to active PPARα. The results showed that chenodeoxycholic acid suppressed WY-14643-induced PPARα activation, whereas WY-14643 suppressed chenodeoxycholic acid-induced FXR activation. These suppressive effects were abolished by using HX531, an RXRα inhibitor. The results suggest a crosstalk between PPARα and FXR potentially through RXRα competition. However, PPARα agonists can also cause liver injury (Hedrington and Davis, 2018). Although large randomized human trials have shown little or no hepatocellular abnormalities when fibrates were used alone, signs of hepatotoxicity appeared more noticeably when fibrates were combined with other drugs. In some case reports, liver damage was demonstrated with the treatment of fibrates as indicated by increased aminotransferase levels (Ho et al., 2004; Dohmen et al., 2005). The liver function usually improved after discontinuation of treatment with fibrates. Therefore, the aminotransferase levels should be monitored when fibrates are used to treat disease.

## Peroxisome proliferator-activated receptor alpha as a therapeutic target in cholestasis treatment

**FIGURE 3:**
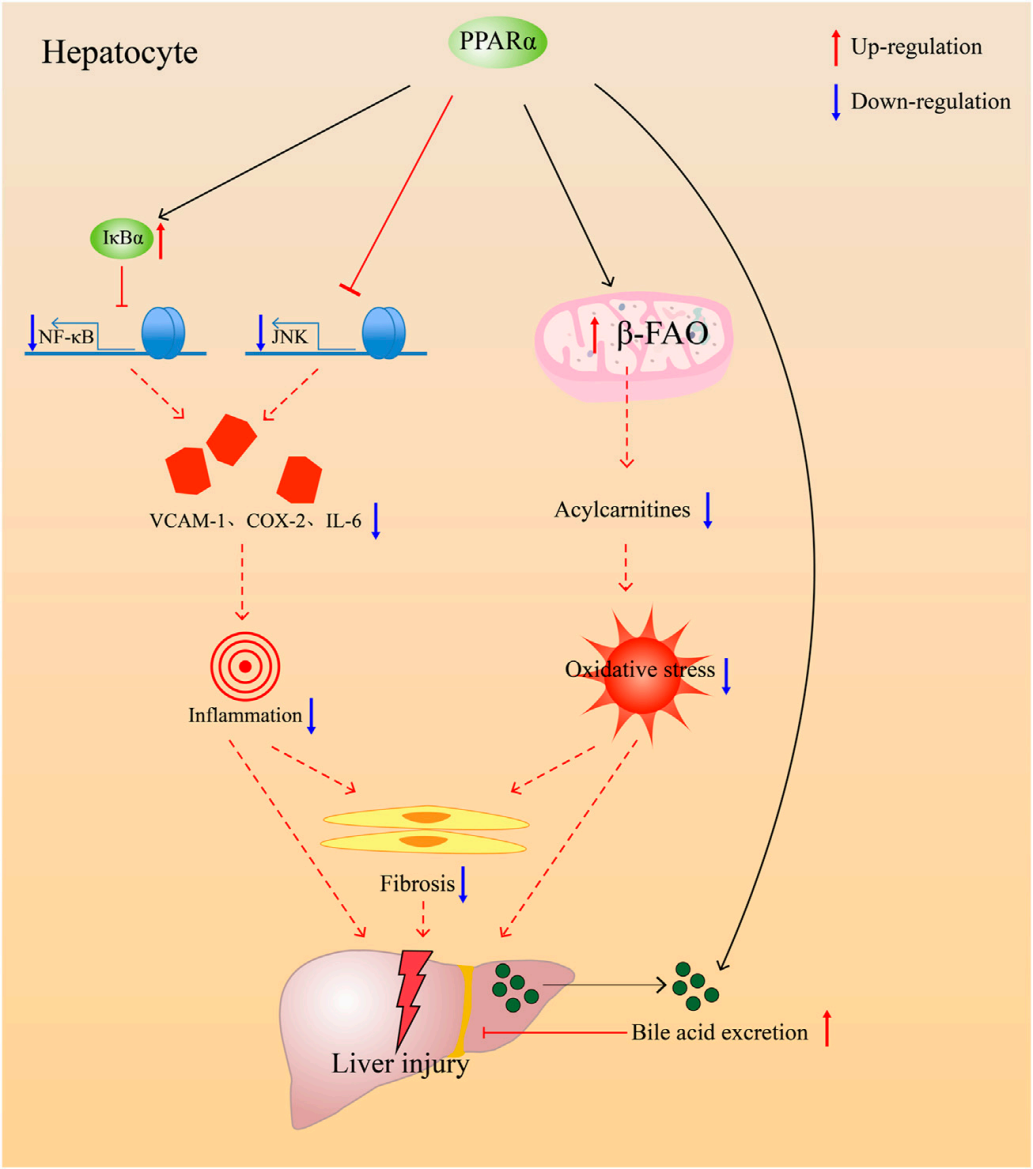
PPARα alleviates injury accused by cholestasis through anti-inflammation, anti-oxidative, antifibrosis, and prevent bile acids accumulate in the liver though promoting bile acids excretion.

The mechanism by which PPARα facilitates cholestasis treatment mainly involves the reduction of bile acid pool size in the liver and regulation of damage due to cholestasis (Figure 3). PPARα agonists are usually used for patients who do not respond to UDCA. The compounds under investigation are shown in Table 2. In general, PPARα agonists are usually used as combined medication with UDCA. They are promising drugs for patients with incomplete biochemical responses to UDCA and those with liver fibrosis and dyslipidemia.

**TABLE 2:** The agonists of PPARα under investigation.

Author	Disease	Drug (daily dose)	Drug combination	Treatment time (months)	Outcome	Side effect
Gallucci et al. (2021)	PBC and PSC	Fenofibrate (145–160 mg)	UDCA	1–53	ALP↓, AST↓, ALT↓, total serum BAs↓, serum BA‐glucuronides↑	None found
Sorda et al. (2021)	PBC	Bezafibrate (400 mg)	UDCA	60	ALP↓, AST↓, ALT↓, GGT↓, improvement of cirrhosis and fibrosis	None found
Reig et al. (2018)	PBC	Bezafibrate (400 mg)	UDCA	38	ALP normalization, jaundice↓, pruritus↓, liver stiffness↓	Transitory myalgi
Le moinne et al. (2018)	PSC	Fenofibrate (200 mg) or bezafibrate (400 mg)	UDCA	6	ALP↓, pruritus↓	None found
Tanaka et al. (2015)	PBC	Bezafibrate (400 mg)	UDCA	24	ALT normalization	None found
Hosonuma et al. (2015)	PBC	Bezafibrate (400 mg)	UDCA	110	ALP↓, Mayo risk score↓	Renal dysfunction, muscle pain
Lens et al. (2014)	PBC	Bezafibrate (400 mg)	UDCA	12	ALP↓, GGT↓, ALT↓, cholesterol↓, triglyceride↓, pruritus↓	Gastrointestinal discomfort, nausea, heartburn
Dohmen et al. (2013)	PBC	Fenofibrate (80 mg); Bezafibrate (400 mg)	UDCA	12	ALP↓, GGT↓, TG↓, LDL↓	None found
Han et al. (2012)	PBC	Fenofibrate (200 mg)	UDCA	3	ALP↓, GGT↓, TG↓, ALT↓, AST↓	None found
Levy et al. (2011)	PBC	Fenofibrate (160 mg)	UDCA	12	ALP↓, AST↓	Heartburn
Takeuchi et al. (2011)	PBC	Bezafibrate (400 mg)	UDCA	24	ALP↓	None found
Liberopoulos et al. (2010)	PBC	Fenofibrate (200 mg)	UDCA	2	ALP↓, GGT↓, ALT↓, cholesterol↓, TG↓	None found

### Anti-cholestasis

In some studies, researchers usually gavage mice with ANIT and lithocholic acid to injure normal hepatocytes or block bile flow with BDL to create a pathological model similar to cholestasis. The transporter-related gene knockout technique has been used in conventional cholestasis models. Fenofibrate, a PPARα agonist, has an effective anti-cholestasis function. In a rat model of BDL, fenofibrate induced a decrease in serum biochemical index and eased hepatocellular damage by increasing PPARα expression within liver cells (Cindoruk et al., 2007). In another rat model of ANIT, the protective effects of fenofibrate against cholestasis-induced hepatic injury depended on PPARα and fenofibrate doses (Dai et al., 2017). When subjected to cholic acid dietary challenge, PPARα^−/−^ mice showed bile acid accumulation in their livers, resulting from decreasing levels of mRNA-encoding transporters, including Abcb11, Abcb4, Abca1, Abcg5, and Abcg8 (Li et al., 2012). In the rat primary biliary cirrhosis model, a novel PPARα/δ dual agonist 5c demonstrated excellent *in vivo* efficacy (Jiang et al., 2019). Fibrate drugs are PPARα agonists and are mainly used as cholesterol-lowering drugs for patients with elevated triglycerides. Fibrate treatment is effective for PBC patients with incomplete response to UDCA (Cuperus et al., 2014). In patients with UDCA-refractory PBC, additional fibrate treatment is associated with the normalization of ALP, lowered risk of cirrhosis development, and reduced risk of hepatic deterioration (Chung et al., 2019). Fenofibrate, a member of the fibrate family, is a widely used alternative therapy for cardiovascular diseases. It contains high-affinity PPARα agonists (Issemann and Green, 1991). In a pilot study, fenofibrate was added to 22 patients with partial response to UDCA (Han et al., 2012). The results showed that fenofibrate effectively improved the liver biochemical test results in patients who had a partial response to UDCA monotherapy, and no obvious adverse effects were observed in patients who received fenofibrate (Han et al., 2012). Recently, researchers assessed fenofibrate, a PPARα agonist, as a combination therapy drug with UDCA in patients with PBC or PSC who had insufficient biochemical responses to UDCA (Ghonem et al., 2020). The addition of fenofibrate significantly reduced serum ALP levels by 82% vs. those receiving ursodiol monotherapy and normalized serum ALP values by 84% in all patients. Meanwhile, the production of proinflammatory cytokines was suppressed with the addition of fenofibrate. Thus, PPARα agonists can be used in treating cholestatic liver disorders.

### Anti-inflammation

Liver injury often accompanies cholestasis and causes cholestasis to further deteriorate into other diseases. Inflammation and oxidative stress are the common causes of liver damage in cholestasis. As an organ of the immune system, the liver induces harmful liver inflammation when suffering from viral infection (Yang et al., 2019). Cholestasis is often accompanied by inflammation characterized by neutrophil infiltration (Wu et al., 2003). Neutrophils are found in the blood and are attracted to the site of inflammation by chemotactic substances when inflammation occurs. ICAM-1 is a protein responsible for the accumulation of neutrophils in the liver (Jaeschke, 1997). In patients with extrahepatic cholestasis, ICAM-1 expression and neutrophil recruitment are upregulated in the liver during extrahepatic cholestasis, which may lead to inflammatory damage to the liver (Gulubova, 1998). Other research also revealed that ANIT drives toxicity toward hepatocytes through neutrophils (Kodali et al., 2006). Thus, the treatment of inflammation is also an important part of the treatment of cholestatic liver injury.

In addition, PPARα can regulate inflammation in the body to regulate lipid and glucose homeostasis (Han et al., 2020). Exercise is a protective factor for lower levels of local inflammatory markers and less myocardial apoptosis, and it seems to be related to the presence of PPARα (Santos et al., 2016). When PPARα^−/−^ mice were treated with proinflammatory substances, they suffered more injury from inflammation due to inflammasome activation or an increase in TNF-α level in the body (Li et al., 2012; Batatinha et al., 2017; Gugliandolo et al., 2019). In addition to this, fibrates had been proved to have the ability to treat neuroinflammation caused by paclitaxel (Caillaud et al., 2021). Therefore, we can conclude that PPARα agonists have a therapeutic effect on inflammation. NF-κB proteins are the key regulators of innate and adaptive immune responses, which can accelerate cell proliferation, inhibit apoptosis, promote cell migration and invasion, and stimulate angiogenesis and metastasis (Taniguchi et al., 2018). NF-κB causes inflammation by promoting ICAM excretion and enabling neutrophils to aggregate. PPARα activator has been reported to have anti-inflammatory properties (Huang et al., 2007; Hennuyer et al., 2016; Huang et al., 2016). WY-14643 can inhibit endotoxin-induced inflammation by suppressing the mRNA expression of IL-6, IL-1β, and TNF-α via controlling the NF-κB pathway (Huang et al., 2016). The same effect has been observed in human epithelial cells (Marx et al., 1999). The absence of PPARα may induce the overexpression of proinflammatory cytokines in LPS stimulus, which can further indicate its effect on anti-inflammation. PPARα treats inflammatory disease by promoting cell autophagy and inhibiting the inflammatory response (Jiao et al., 2016). PPARα induces the expression of the inhibitory protein NF-kappa-B-inhibitor alpha (IkBa) in human aortic smooth muscle cells, as well as in primary human hepatocytes; then, it inhibits NF-κB activation to decrease inflammation (Delerive et al., 2000). Fenofibrate, a PPARα agonist, provides protection against hepatic injury by inhibiting the JNK and NF-κB signaling pathways (Dai et al., 2017). PPARα can promote the inactivation of NF-κB during the inflammatory reaction, and the inhibition can inhibit the inflammatory cascade (Korbecki et al., 2019). In addition to inflammation, bile acids in the liver can cause mitochondrial damage and oxidative stress. PPARα activation by fenofibrate provides protection against liver damage by recovering mitochondrial fatty acid β-oxidation (β-FAO) which impaired by ANIT (Zhao et al., 2017). Therefore, PPARα eliminates oxidative stress by increasing the expression of β-FAO. Moreover, ANIT-induced liver fibrosis was alleviated by fenofibrate through PPARα (Lu et al., 2021), and anti-inflammation and antioxidation may play important roles in antifibrosis (Chung et al., 2018).

This mechanism of PPARα protection against inflammation may offer additional therapeutic opportunities for cholestatic liver diseases. Meanwhile, antifibrosis and antioxidant stress are important to the improvement of liver injury. Recently, PPARα has been found to be related to liver regeneration in mice (Fan et al., 2021). This function of liver regeneration may be a promising way to improve the condition of patients with cholestasis. The therapeutic effect of PPARα on inflammation and the regulation of bile acid homeostasis offers additional therapeutic opportunities for the treatment of cholestatic liver diseases.

## Conclusion

With the advanced understanding of the pathology of cholestasis, liver injury has been found to have various causes. Besides bile acid directly damaging hepatocytes, inflammation and oxidative stress can also cause liver injury. Meanwhile, inflammation also affects bile acid transporter proteins. We can speculate that treating inflammation is as important as the regulation of bile acid homeostasis in the therapy of cholestasis. At present, cholestasis is mostly treated by regulating bile acid, and there are certain side effects. The main physiologic function of PPARα is to control glucose metabolism and energy combustion. However, PPARα is involved in the control of bile acid homeostasis, and the treatment of inflammation during cholestasis provides us new perspective to treat this disease. Therefore, finding safe and effective PPARα activators may have important clinical significance for the amelioration of cholestasis (Table 3).

**TABLE 3:** Category of experiments.

Section of the article	Category of experiments	Ways	Outcome
Section 2	Animal (mice)	BDL	Liver injury occurs with neutrophil accumulation
Section 2	Clinical (PBC patient)	Detect inflammatory cytokines of patients with PBC	All major pro-inflammatory cytokine levels are enhanced in PBC patients
Section 2	Animal (rat)	Dosing corilagin	Corilagin reduced cholestatic liver injury by anti-inflammation effects
Section 2	Animal (mice)	Inject rotavirus	Inflammation decreases the levels of liver transporter
Section 4.1	Animal (mice)	Bile acid-enriched diet	PPARα was inhibited by bile acids
Section 4.1	Animal (rat)	Bile acid-enriched diet	PPARα and its target protein was inhibited by bile acids
Section 4.2	Animal (mice)	Dosing ciprofibrate	Ciprofibrate decreased mRNA of CYP7A1
Section 4.2	Cell (HepG2)	Cultivating with WY-14643	Agonist of PPARα reduced the availability of HNF-4
Section 4.2	Animal (mice)	Dosing Perfluorodecanoic acid	Agonist of PPARα reduced the mRNA level of Cyp7a1, Cyp8b1 and NTCP
Section 4.2	Animal (mice)	Dosing WY-14643	WY-14643 induces the expression of CYP3A4
Section 4.2	Animal (mice)	Knockout	The expression of Cyp8b1 decreased in stard10^−/−^ mice with damage of PPARα activity
Section 4.2	Cell (HepG2)	Cultivating with WY-14643	WY-14643 treatment activated CYP8B1
Section 4.2	Animal (rat)	Dosing clofibrate	Agonist of PPARα active the mrna expression of OATP
Section 4.2	cell (HepG2)	Cultivating with WY-14643	WY-14643 increases UGT2B4 mRNA levels
Section 4.3	Animal (mice)	Dosing ciprofibrate	Agonist of PPARα active the mRNA expression of MDR2
Section 4.3	Cell (HepG2)	Cultivating with fenofibrate	Agonist of PPARα active the mRNA expression of MDR3
Section 4.3	Animal (mice)	Dosing clofibrate	Clofibrate reduced the total bile acids through increase in the level of BSEP
Section 4.4	Cell (HepG2)	Cultivating with chenodeoxycholic acid	The increase of hPPARα mRNA levels in a dose-dependent way with chenodeoxycholic acid
Section 4.4	Animal (Pparα^−/−^ mice)	Dosing 0.05% ANIT	The levels of Shp and Fxr mRNA high in Pparα^−/−^ mice than the control group
Section 5.1	Animal (rat with BDL)	Dosing fenobibrate	A decrease in serum biochemical index and eased hepatocellular damage
Section 5.1	Animal (rat primary biliary cirrhosis model)	PPAR alpha/delta dual agonist	Improve the pathological condition of rats
Section 5.1	Clinical (PBC patient)	Additional fibrate treatment	Normalization of ALP, lowered risk of cirrhosis development
Section 5.1	Clinical (PBC patient)	Additional fenofibrate treatment	Improving liver biochemical tests
Section 5.1	Clinical (PBC and PSC patient)	Additional fenofibrate treatment	Reduced serum ALP levels
Section 5.2	Animal (mice)	Fenofibrate (i.p.)	Decrease neuroinflammation involves the regulation of PPAR-⍺ expression
Section 5.2	Cell (synovial fibroblasts.)	Cultivating with WY-14643	WY-14643 greatly inhibited the production of pro-inflammatory cytokines
Section 5.2	Cell (endothelial cell)	Cultivating with WY-14643 or fenofibrate	PPAR alpha activators inhibited TNF-alpha-induced VCAM-1
Section 5.2	Animal (rat)	Dosing fenobibrate	PPAR alpha activators inhibited liver damage through recovering β-FAO
Section 5.2	Animal (mice)	Dosing fenobibrate	Feno fibrate reverses cholestatic liver fibrosis

**FIGURE 4:**
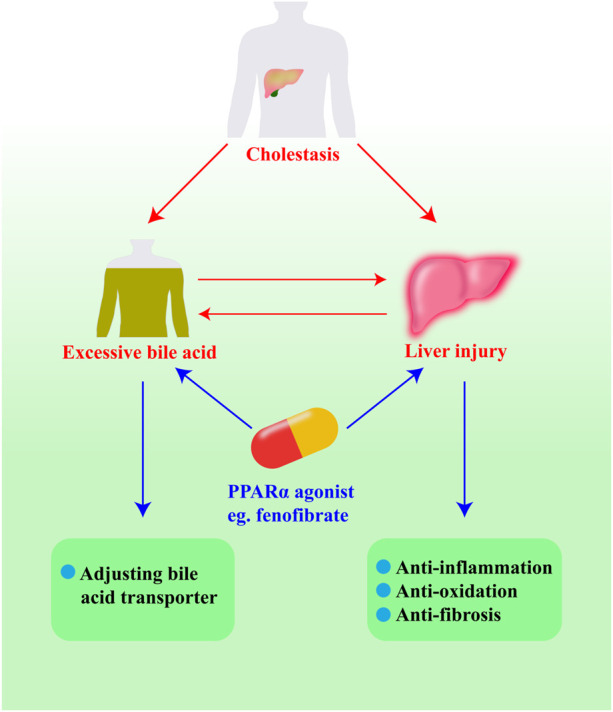
The pathways of PPARα agonists protecting against cholestasis.

As shown in the scheme (Figure 4), we introduce the ways in which PPARα regulates bile acid homeostasis and reduces liver injury. Through activating bile acids or cholesterol, PPARα can increase the expression of bile acid transporter proteins and bile acid detoxification proteins, including CYP7A1, BSEP, MDR3, MRP2, MRP3, MRP4, CYP3A4, UGTs, and SULTs. Meanwhile, PPARα regulates inflammatory factors such as TNF-α, IL-1β, MCP-1, and MIP-2. It also regulates the activation of neutrophils by inhibiting the expression of JNK and NF-κB. PPARα can also control enzymes to inhabit β-FAO, which may lead to liver injury by means of oxidative stress. In addition, natural PPARα activators are necessary for the treatment of cholestasis as they can suppress hepatocyte apoptosis, necrosis, and fibrosis.

With the progress of science and technology, there is a deeper understanding of the pathologic mechanism of cholestasis. The comprehensive regulation of bile acids and liver injury undoubtedly plays a role in treating the symptoms and root causes of cholestasis. However, the regulation of PPARα in cholestasis, including the crosstalk of PPARα and FXR, is still unclear. Due to the existence of species specificity, some experimental results may not completely correspond to the findings in clinical settings. Further studies are needed to improve our knowledge behind the PPARα mechanism. At present, fibrates combined with other therapeutic drugs seem to be a possible therapy for cholestatic liver injury in the clinic. PPARα activators are promising in the treatment of cholestasis.
